# Biological therapy for ulcerative colitis

**DOI:** 10.1093/gastro/gou070

**Published:** 2014-10-24

**Authors:** Zubin Arora, Bo Shen

**Affiliations:** ^1^Department of Internal Medicine, the Cleveland Clinic Foundation, Cleveland, OH, USA and ^2^Department of Gastroenterology and Hepatology, the Cleveland Clinic Foundation, Cleveland, OH, USA

**Keywords:** ulcerative colitis, biological therapy, TNFα antagonists, anti-integrin agents

## Abstract

Ulcerative colitis (UC) is a major form of inflammatory bowel disease (IBD) worldwide. Better understanding of the pathogenesis of UC has led to the development of novel therapeutic agents that target specific mediators of the inflammatory cascade. A number of biological agents have been approved by the U.S. Food and Drug Administration (FDA) for the treatment of UC and several more are currently in various phases of drug development. The commonly used agents include TNFα antagonists (e.g. infliximab, adalimumab, and golimumab) and anti-integrin agents (vedolizumab). These biological agents have profoundly influenced the management of UC patients, especially those with refractory disease. This paper reviews the currently available knowledge and evidence for the use of various biological agents in the treatment of UC.

Ulcerative colitis (UC) is a major form of inflammatory bowel disease (IBD), characterized by chronic inflammation involving the colon and rectum. It is a cause of significant morbidity worldwide and its incidence and prevalence appear to be increasing with time [1]. Patients with UC frequently experience episodes of bloody diarrhea with or without mucous, abdominal pain, fever and weight loss. The pathogenesis of UC is believed to involve a dysregulated immune response to an unknown environmental stimulus within the large bowel [2]. A number of pro-inflammatory cytokines, including interleukin-4 (IL-4), IL-5, IL-6, IL-10 and tumor necrosis factor-alpha (TNF-α), have been recognized to play a central role in mediating this immune response [2–4]. The knowledge in the pathogenetic pathways has resulted in the development of several novel therapeutic agents for UC that target these pro-inflammatory molecules in order to contain the inflammatory cascade or pathways. Therapy that is directed at a specific mediator of inflammation has been termed ‘biological therapy'. Unlike conventional drugs, which tend to suppress the entire immune system, the mechanism of action of biological agents is more selective.

Among the pro-inflammatory cytokines, the role of TNF-α has been the most extensively studied. Excessive production of TNF-α from activated macrophages and T-lymphocytes leads to further activation of macrophages and T-lymphocytes, expression of adhesion molecules on vascular endothelium and recruitment of neutrophils, resulting in a vicious cycle of increasing inflammation [3, 5, 6]. Elevated levels of TNF-α have been observed in the colon tissue specimen, as well as in the blood, stool, and urine samples of patients with UC [7–9]. As a result, TNF-α has been an attractive target for biological agents. Three anti-TNF agents have been developed and some have been approved by the U.S. Food and Drug Administration (FDA) for the treatment of both UC and Crohn’s disease (CD).

Despite promising results with the use of anti-TNF agents in the management of UC, some patients continue to require corticosteroids or develop primary or secondary non-response to anti-TNF therapy. Failure to respond to biological therapy is categorized into two types: primary and secondary failure. In patients with the primary failure, no improvement in clinical signs and symptoms is noted with initial induction therapy using the treatment agent. In contrast, patients with the secondary failure exhibit initial improvement with induction therapy and subsequent maintenance therapy but eventually lose the response. As a result, several other ‘key players' of inflammatory pathways have been investigated as therapeutic targets. These include adhesion molecules, which help in migration of leukocytes (e.g. integrin, intercellular adhesion molecule [ICAM], vascular cell adhesion molecule [VCAM]), other cytokines (like IL-12, IL-23 and IL-10) and T-lymphocyte receptors (such as CD28 and CD25).

This review provides updated information on various biological agents in the treatment of UC.

## Biological therapy for induction and maintenance of remission in ulcerative colitis

### Anti-TNF agents

The widespread use of anti-TNF-α agents has changed the treatment paradigm in the management of patients with UC, as well as in those with CD. Several strategies for targeting and inhibiting TNF-α have been developed and include neutralization of TNF-α with monoclonal antibodies and inhibition of its production or its receptors [3]. Four agents are currently used in the clinical treatment of IBD. Three of these—infliximab, adalimumab, and golimumab—have been approved by the FDA for the treatment of UC patients.

#### Infliximab

Infliximab was the first agent to receive FDA approval for induction and maintenance therapy in UC [10]. It is a chimeric, monoclonal antibody against TNF-α, which binds to both soluble and membrane-bound forms of TNF-α [4]. It is only available for administration as an intravenous infusion.

The efficacy of infliximab in the treatment of UC has been demonstrated in both open-label and randomized, controlled trials [10–20]. Two large, multi-center, randomized, double blinded, placebo-controlled trials—the Active Ulcerative Colitis Trials 1 and 2 (ACT 1 and ACT 2, respectively)—have provided evidence of the efficacy of infliximab in UC patients [10]. Both of these trials included 364 ambulatory patients with moderate-to-severe UC (defined as a total Mayo Score of 6–12 and a Mayo Endoscopy Subscore of ≥2) who had failed to respond to—or were intolerant to—conventional treatment with corticosteroids or immunomodulators (azathioprine or 6-mercaptopurine) [21]. ACT2 also included patients previously treated with 5-aminosalicylate agents. Patients were randomized to receive either placebo or infliximab infusion (5 or 10 mg/kg) at weeks 0, 2 and 6, and then every 8 weeks until week 46 in ACT1 and week 22 in ACT2. Clinical response was defined as a reduction in the Mayo Score by at least 3 points and at least 30%. Clinical remission was defined as a total Mayo Score of ≤2 points with no sub-score exceeding 1. The total duration of follow-up was 54 weeks in ACT1 and 30 weeks in ACT2. The patients in the infliximab group experienced significantly higher rates of clinical response and clinical remission at weeks 8, 30 and 54 than the placebo group in both clinical trials. The rates of adverse events in the infliximab and placebo groups were comparable.

The efficacy of infliximab increases further when used in combination with azathioprine. In a recent double-blinded, placebo-controlled trial, 239 anti-TNF-α therapy-naïve patients with moderate-to-severe UC were randomized to receive either (i) intravenous infusions of infliximab (5 mg/kg dose) at weeks 0, 2, 6, and 14, plus daily oral placebo capsules or (ii) oral azathioprine (2.5 mg/kg daily) plus placebo infusions on the infliximab schedule or (iii) combination therapy with both drugs [22]. Significantly more patients achieved corticosteroid-free clinical remission in the infliximab + azathioprine group, than either therapy alone.

A meta-analysis of five randomized, controlled trials, examining the efficacy of infliximab in the treatment of moderate-to-severe UC, showed that infliximab therapy was associated with a higher rate of induction of remission than placebo [23].

Infliximab has also been used for the treatment of chronic pouchitis, which is a common complication after proctocolectomy with ileal pouch anal anastomosis surgery for UC; however, experience with the use of infliximab in this setting is currently limited to case reports and series [24–31]. Although results from these studies suggest a promising role for infliximab, further data from larger randomized trials are need to demonstrate its guaranteed efficacy in pouchitis patients.

#### Adalimumab

Adalimumab is a fully humanized monoclonal antibody against TNF-α which is administered subcutaneously. It was approved by the FDA in 2012 for the treatment of moderate-to-severe UC in adults after its efficacy was demonstrated in several randomized and non-randomized studies [32–37]. Because of the subcutaneous route of administration, it can be self-administered by patients at home, thus avoiding the high cost and inconvenience of hospital visits for intravenous infusions.

Two large, randomized, controlled trials demonstrated the efficacy of adalimumab for the induction and maintenance of remission in UC patients. These were the UC Long-Term Remission and Maintenance with Adalimumab 1 and -2 trials (ULTRA 1 and ULTRA 2, respectively) [35, 38].

ULTRA 1 was an 8-week, multicenter, randomized, double-blinded, placebo-controlled trial investigating the use of adalimumab as induction therapy in patients with moderate-to-severe UC despite conventional therapy [35]. In this trial, 576 patients were randomized to receive either placebo or one of two different regimens of adalimumab: (i) regimen 160/80: adalimumab 160 mg at week 0; 80 mg at week 2; 40 mg at weeks 4 and 6 or (ii) regimen 80/40: adalimumab 80 mg at week 0; 40 mg at weeks 2, 4 and 6, while concurrently receiving stable treatment with oral corticosteroids or immunomodulators. At the end of 8 weeks, patients receiving adalimumab 160/80 were nearly twice as likely to achieve clinical remission as those receiving placebo (18.5% *vs.* 9.2%; *P = *0.031). There was no significant difference in remission rates between patients receiving adalimumab 80/40 and placebo (10% *vs.* 9.2%; *P = *0.833). Simultaneously, patients receiving adalimumab 160/80 showed significantly greater improvements in nutrition and inflammatory markers than the placebo group.

This study was followed by ULTRA 2, which was a 52-week study assessing the efficacy of adalimumab as maintenance therapy in UC patients [38]. This trial included 494 patients with moderately-to-severely active UC for the last 3 months despite concurrent treatment with corticosteroids or immunomodulator drugs. Patients were also stratified, based on whether they had previously been treated with TNF-antagonists or were anti-TNF therapy-naïve. The patients were randomized to receive either placebo or adalimumab (160 mg at week 0; 80 mg at week 2 and then 40 mg every 2 weeks starting week 4). A significantly higher proportion of patients in the treatment group achieved clinical remission at 8 weeks and remained in clinical remission at the end of 52 weeks, than was seen in the placebo group. Clinical response rates were also higher at weeks 8 and 52 in the treatment group, as compared with the placebo group. Importantly, on sub-group analysis, adalimumab was found to be effective at week 8 only in the anti-TNF therapy-naïve patients. In patients who had received anti-TNF therapy prior to their participation in the trial, clinical remission rates at week 8 were similar in the treatment and placebo arms.

An open-label extension study to ULTRA 1 and 2 is currently under way, to assess the long-term efficacy of adalimumab in UC patients [39]. Preliminary results suggest that the efficacy of adalimumab was sustained for up to 3 years for the treatment of moderate-to-severe UC.

#### Golimumab

Golimumab is a fully humanized monoclonal antibody against TNF-α and is the latest anti-TNF agent to receive FDA approval for treatment of moderate-to-severe ulcerative colitis. Similarly to adalimumab, it is administered subcutaneously and can therefore be self-administered by patients at home. Data regarding the efficacy of golimumab in UC comes mainly from two large, double-blinded, randomized, controlled trials, the Program of Ulcerative Colitis Research Studies Utilizing an Investigational Treatment, which was divided into Subcutaneous and Maintenance phases (PURSUIT-SC and PURSUIT-M, respectively) [40, 41]. The PURSUIT-SC trial investigated the efficacy of golimumab as induction therapy, whereas PURSUIT-M studied its efficacy as maintenance therapy for moderate-to-severe UC.

PURSUIT-SC was an integrated phase-II and phase-III trial, in which 1064 anti-TNF therapy-naïve patients with moderate-to-severe UC (Mayo Score: 6–12; Mayo Endoscopic Subscore: ≥2) despite conventional therapy were included [40]. Of these, 774 were in phase III. The phase II trial data indicated that higher doses of golimumab [(i) 200 mg followed by 100 mg and (ii) 400 mg followed by 200 mg given subcutaneously 2 weeks apart] were associated with a higher rate of clinical response and remission than lower doses (100 mg followed by 50 mg given subcutaneously 2 weeks apart). There was no significant difference in the rate of adverse events between the different dose regimens. After the dose-finding phase-II trial, 774 patients were randomized to receive subcutaneous doses of (i) golimumab 400 mg followed by 200 mg, (ii) golimumab 200 mg followed by 100 mg or (iii) placebo at weeks 0 and 2, respectively. The primary and secondary endpoints were measured at week 6 and were defined similarly to the ACT1 and ACT2 trials [10]. The results of the study showed that patients treated with golimumab, with both the 400/200 mg and 200/100 mg regimens, exhibited higher clinical response and clinical remission rates than with placebo. The rates of adverse events were significantly lower in the golimumab arm than that in the placebo arm.

The subsequent PURSUIT-M trial included the same participants as PURSUIT-SC, to study the efficacy of golimumab as maintenance therapy [41]. In PURSUIT-M, 464 patients who had responded to golimumab induction therapy in PURSUIT-SC were randomized to receive placebo or golimumab (50 mg or 100 mg) every 4 weeks for 52 weeks. Patients who had responded to placebo continued to receive it and all non-responders were given 100 mg golimumab. After 54 weeks, patients receiving golimumab experienced higher rates of clinical response than patients who received placebo. In addition, patients who received a 100 mg dose of golimumab had significantly higher rates of clinical remission at 54 weeks, compared with patients receiving placebo or 50 mg dose. Adverse events and infections occurred more frequently in the golimumab group than in the placebo group.

#### Certolizumab pegol

Certolizumab pegol is the first pegylated, humanized monoclonal antibody against TNF-α. In fact, it contains only the antigen binding fragment (Fab) of the monoclonal antibody which has been conjugated to polyethylene glycol and does not contain the crystallizable (Fc) or tail portion of the antibody [42]. These unique characteristics afford some special properties to certolizumab pegol. Firstly, PEGylation of the antibody leads to an increase in its circulating half-life and improvement in bioavailability and pharmacokinetics, thereby permitting a minimum dosing interval of 2 weeks [43]. Also, as a result of PEGylation, the preferential penetration of certolizumab pegol into the inflamed tissue (over the non-inflamed tissue) is increased [44]. Secondly, due to the absence of the Fc portion, certolizumab pegol does not induce complement-dependent cytotoxicity or antibody-dependent cell-mediated cytotoxicity *in vitro* [45]. Lack of the Fc region also prevents trans-placental transfer of certolizumab pegol during pregnancy [46].

Certolizumab pegol has been shown to be effective in the treatment of CD and was approved by the FDA for this indication [42, 47]; however, its efficacy in the management of UC patients is currently under investigation in a phase II clinical trial [48].

### Anti-integrin therapy

Integrins are a group of transmembrane receptors, which are involved in cellular adhesion and signal transduction. Integrins play an important role in the inflammatory response by participating in the adhesion of leukocytes to vascular endothelium and allowing it to migrate across the blood vessels at the inflammatory site [49]. Blocking these cell surface receptors results in decreased migration of leukocytes, thus decreasing the inflammatory response. Novel biological agents, targeting integrins and other cellular adhesion molecules, are currently under development. Natalizumab, the first drug to be developed in this class, was a monoclonal antibody against α4 integrin, which was shown to be effective in the treatment of CD. The use of natalizumab has been associated with an increased risk for progressive multifocal leukoencephalopathy (PML), mainly because it also reduces the migration of leukocytes into the nervous system due to its non-selective binding to α4 integrin [50]. Another drug, vedolizumab, which is more selective for the gut, has recently been shown to be effective for the treatment of UC and CD in large randomized trials and will be discussed further.

#### Vedolizumab

Vedolizumab is a humanized monoclonal antibody that selectively binds to α4β7 integrin, which is located exclusively on the surface of gut-homing leukocytes [50–52]. By binding to the α4β7 integrin, it selectively blocks the adhesion and trans-endothelial migration of leukocytes in the gut without interfering with lymphocyte migration in the nervous system, thereby leading to a reduction in the inflammatory response in IBD patients. It is administered intravenously.

The efficacy of vedolizumab in the treatment of UC has been demonstrated in the GEMINI 1 trial [52]. This was a phase III, multicenter, double-blind, placebo-controlled study that integrated two different trials: for induction and maintenance therapies. The trial of induction therapy included 374 patients who were randomized to receive placebo or vedolizumab (300 mg at weeks 0 and 2). In addition, to meet the sample size requirements of the maintenance trial, a second group of 521 patients was enrolled, who received open-label vedolizumab at weeks 0 and 2, with responders at week 6 subsequently entering into the maintenance trial. All patients had moderate-to-severe UC (Mayo Score: 6–12 with endoscopy sub-score ≥2) and had failed to respond to previous therapy with at least one agent, including corticosteroids, immunomodulators and TNFα antagonists. The results of the induction trial showed that a significantly greater number of patients who received vedolizumab achieved clinical response and clinical remission at the end of 6 weeks, than those with placebo.

For the maintenance trial, patients from either above group who had a response to vedolizumab were randomized to receive either vedolizumab or placebo every 4 or 8 weeks. At the end of 52 weeks, patients receiving vedolizumab were significantly more likely to have clinical remission and clinical response than with placebo, including those who had previously failed anti-TNF therapy. Additionally, patients in the treatment group were less likely to require corticosteroids and had greater improvements in quality of life scores than those in the placebo group. There was no significant difference in efficacy between the two vedolizumab regimens. Also, the rate of adverse events was similar in the treatment and placebo groups and no cases of PML were observed during the 52 week follow-up.

Vedolizumab was recently approved by the FDA for the treatment of adults with moderate-to-severe UC [53]. An open-label study to determine the long-term efficacy and safety of vedolizumab in the treatment of UC and CD is also currently in progress [54].

### Other biological agents

Despite the recent advances, many patients fail to respond to medical therapy with currently available agents. Ongoing research on the pathogenesis of IBD has provided several more attractive therapeutic targets, for which new drugs are currently under development.

#### PF-547659

PF-547659 is an investigational monoclonal antibody against MAdCAM-1 (addressin), which is the extracellular ligand for α4β7 integrin. It inhibits adhesion and trans-endothelial migration of leukocytes by suppressing the interaction of leukocytes with vascular endothelium, similarly to vedolizumab. Favorable results were observed in a preliminary study in UC patients and further trials are in progress [55].

#### Alicaforsen

Alicaforsen is another anti-adhesion molecule that reduces lymphocyte migration. It is an anti-sense oligonucleotide, causing decreased synthesis of endothelial adhesion molecule ICAM-1. A recent meta-analysis of four phase II studies in UC suggests that alicaforsen is effective for the treatment of moderate-to-severe UC, especially in patients with distal disease [56]. In an open-label trial of 12 patients, an enema formulation of alicaforsen was also shown to be effective for the treatment of chronic pouchitis in patients with underlying UC [57].

## Biological therapy for mucosal healing in ulcerative colitis

Although the main focus of treatment of patients with UC has traditionally been the alleviation of symptoms by inducing and maintaining symptomatic remission, there is increasing evidence to suggest that achieving mucosal healing and reduction in endoscopic disease activity may be as critical as improvement in symptoms in optimizing long-term outcomes [58]. Indeed, mucosal healing has been shown to correlate with better long-term remission rates, fewer disease-related complications and better quality of life for patients [59–61].

The efficacy of biological agents in reversing the tissue damage associated with inflammation has been well studied. The efficacy of infliximab in mucosal healing was assessed during the ACT1 and ACT2 trials [10]. Mucosal healing was defined as a Mayo endoscopic subscore of ≤1. The infliximab group exhibited significantly higher rates of mucosal healing than the placebo group at weeks 8 and 30 in both trials and at week 54 in the ACT1 trial (*P* < 0.009 for all comparisons). Similarly, during the ULTRA2 trial, which assessed the efficacy of adalimumab, mucosal healing rates were higher in the treatment group than those in the placebo group both at week 8 (*P = *0.032) and week 52 (*P = *0.009) [35]. Higher rates of mucosal healing were also seen in patients treated with golimumab than with placebo at weeks 6 and 54 in the PURSUIT-SC and PURSUIT-M trials, respectively [40, 41].

Mucosal healing effects have also been demonstrated with the use of vedolizumab. In the GEMINI 1 trial, patients treated with vedolizumab experienced higher rates of mucosal healing than placebo at both week 6 and week 52 [52].

## Biological therapy and the natural history in ulcerative colitis

The natural history of UC is that of a progressive disease leading to high rates of surgery and morbidity [62]. Treatment with biological therapy has demonstrated the potential to alter the natural history of UC, including reduced colectomy rates and improved quality of life. This may be related to the mucosal healing effects of biological therapy as discussed above. Previous studies have suggested that improvement in mucosal healing rates is associated with better long-term outcomes and reduced rates of colectomy [59, 60].

Using data from the ACT1 and ACT2 trials, a subsequent study demonstrated that patients treated with infliximab had a significantly lower rate of colectomy by week 54 than those treated with placebo [63]. Additionally, patients treated with infliximab had a significantly lower rate of UC-related hospitalizations, surgeries/procedures per 100 patient-years than those treated with placebo.

In another randomized, double-blinded, placebo-controlled trial of 45 hospitalized patients with intravenous steroid-refractory flare of moderate-to-severe UC, patients who received a single infusion of infliximab after administration of steroids required colectomy significantly less frequently at 3 months following the infusion, than those who received placebo [11]. A follow-up study on the same cohort also showed similar results at 3-year follow-up [64].

Similar results have been demonstrated with use of adalimumab. Using data from the ULTRA 2 trial, subsequent studies have indicate that adalimumab therapy is associated with significantly reduced risk of hospitalization and significantly greater improvement in health-related quality of life measures during 52 weeks than with placebo [65, 66].

## Adverse effects of biological therapy

The randomized, controlled trials have demonstrated a generally favorable safety profile for biological agents; however, a few patients do experience side-effects and biological agents have been occasionally associated with severe—sometimes life threatening—adverse effects necessitating careful monitoring of therapy [67]. Many of these adverse effects are related to the immunosuppressive effects of biological agents. These are discussed below.

### Infections

Patients receiving biological therapies are at an increased risk of opportunistic infections and re-activation of latent infections, such as tuberculosis and hepatitis B virus. This risk is further increased by combination therapy with other immunomodulators [68]. As mentioned earlier, natalizumab has been associated with increased risk of PML caused by John Cunningham (JC) virus. Current guidelines recommend routine screening for latent tuberculosis and hepatitis B virus infection prior to initiation anti-TNF therapy [69, 70]. For the same reason, live attenuated vaccines should not be administered to patients receiving TNF-therapy, or those who have discontinued anti-TNF therapy in the last 3 months [71].

### Neoplasms

Biological agents are also associated with an increased risk of malignancies, especially lymphoma [72]. This is probably due to the inhibition of the apoptotic and tumor suppressive functions of TNF-α. A current malignancy or history of lymphoma is a contraindication for anti-TNF therapy, and expert advice from an oncologist should be sought in the case of a prior malignancy [68]. Increased awareness and vigilance is important for the early recognition of these complications.

### Others

Many of the biological agents are immunogenic and patients frequently develop antibodies against these drugs, which can interfere with their therapeutic effects. This problem is more frequent with chimeric agents like infliximab, which are considered more immunogenic than with fully humanized agents like adalimumab [67]. Also, patients receiving biologics frequently develop auto-antibodies, the clinical significance of which remains unclear [73]. Patients may also develop infusion and injection site reactions which, in rare occasion, may be severe enough to warrant discontinuation of therapy or switching to a different agent [74]. Other adverse effects include worsening of congestive heart failure and eczematous skin lesions, which are class effects of anti-TNF agents [68].

## Conclusions

A number of biological agents are currently available for the treatment of patients with IBD (Table 1). These agents have been shown to be effective in the induction and maintenance of remission in patients with UC. With advances in the understanding of pathogenesis of UC, an increasing number of therapeutic agents are currently being developed (Table 2). These agents will provide new opportunities for treatment of patients with refractory disease and add to the armamentarium of the treating physicians.

**Table 1.** Biological agents approved by the FDA for use in ulcerative colitis.

Biological agent	Mechanism of action	Structure	Route of administration	Year of approval
Infliximab	Anti-TNFα	25% murine, 75%human IgG1	Intravenous	2005
Adalimumab	Anti-TNFα	Human IgG1	Subcutaneous	2012
Golimumab	Anti-TNFα	Human IgG1	Subcutaneous	2013
Vedolizumab	Anti-α4β7 integrin	Humanized IgG1	Intravenous	2014

**Table 2.** Biological agents currently under investigation for use in ulcerative colitis.

Biological agent	Mechanism of action	Structure	Route of administration	Clinical trials
Certolizumab pegol	Anti-TNFα	Pegylated humanizedFab’ fragment	Subcutaneous	NCT01090154
PF-547659	Anti-MAdCAM-1	Human IgG2	Subcutaneous	NCT01620255, NCT01771809
Alicaforsen	Decreased synthesisof ICAM-1	Antisense oligonucleotide	Enema, intravenous	NCT00063414, NCT00063830

## Funding

The research and education activity of Bo Shen is supported by the Ed and Joey Story Endowed Chair.

*Conflict of interest statement:* none declared.
